# Supplementary material for: The genome sequence of a cluster fly, Pollenia griseotomentosa (Jacentkovský, 1944)
Source: Wellcome Open Res. 2025 Feb 3;10:42. [Version 1] doi: 10.12688/wellcomeopenres.23625.1 (PMC12280869; doi:10.12688/wellcomeopenres.23625.1)
Supplement: Peer review history

# Reviewer report: Reviewer response for version 1

**DOI:** 10.21956/wellcomeopenres.26065.r126012

**Published:** 2025-08-12

**Authors:** Arun Arumugaperumal

**Affiliations:** 1 Department of Biotechnology, Rajalakshmi Engineering College, Rajalakshmi Group of Institutions, Chennai, India

This is the first report of the genome sequence of *Pollenia griseotomentosa*. The photograph of the organism was clearly documented in figure 1. Adding a common object for reference scale would improve the quality of this figure greatly. The genome sequence assembly reported is of size 1046.3 Mb. The number of chromosomes was reported as 6 and is coinciding with that of other Pollenia genomes reported before. The mitogenome has also been separately presented. Cluster flies are parasites of earthworms. The genome sequence presented here may help in controlling the pest using biotechnological interventions. The article may be indexed in its present form.

Are sufficient details of methods and materials provided to allow replication by others?

Yes

Is the rationale for creating the dataset(s) clearly described?

Yes

Are the datasets clearly presented in a useable and accessible format?

Yes

Are the protocols appropriate and is the work technically sound?

Yes

Reviewer Expertise:

Bioinformatics; Genomics

I confirm that I have read this submission and believe that I have an appropriate level of expertise to confirm that it is of an acceptable scientific standard.

# Reviewer report: Reviewer response for version 1

**DOI:** 10.21956/wellcomeopenres.26065.r126017

**Published:** 2025-07-21

**Authors:** Komal Kumar Bollepogu Raja

**Affiliations:** 1 Baylor College of Medicine, Houston, Texas, USA

Sivell O. et al. present the genome sequence of *Pollenia griseotomentosa *with a total length of 1046.3 megabases. The phylogeny, morphology, and the ecological asepcts of this organism has been sufficiently introduced in the background. The data quality is impressive with low number of scaffolds and great contig assembly metrics, BUSCO score, k-mer completeness. The Hi-C contact map shows a clear diagonal signal. Together, these data suggest that the genome build is high quality. The experimental methods and bioinformatics tools utilized are standard and allow for reproducibility. Overall, the manuscript is well written and the data is a valuable resource for comparative genomics studies.

Are sufficient details of methods and materials provided to allow replication by others?

Yes

Is the rationale for creating the dataset(s) clearly described?

Yes

Are the datasets clearly presented in a useable and accessible format?

Yes

Are the protocols appropriate and is the work technically sound?

Yes

Reviewer Expertise:

Genomics, Bioinformatics, single cell genomics and Neurobiology

I confirm that I have read this submission and believe that I have an appropriate level of expertise to confirm that it is of an acceptable scientific standard.

# Reviewer report: Reviewer response for version 1

**DOI:** 10.21956/wellcomeopenres.26065.r126013

**Published:** 2025-07-21

**Authors:** Jaakko Pohjoismäki

**Affiliations:** 1 University of Eastern Finland, Joensuu, Finland

In the presented manuscript, Olga Sivell, Ryan Mitchell, Steven Falk together with the Darwin Tree of Life Consortium provide a genome report for Pollenia griseotomentosa.

 The genome sequencing, assembly and annotation follow the DToL pipeline and the produced genome assembly is of high quality, meeting the EBP standards. There should be little doubt about the species identity, as also the used COI-barcodes separate P. griseotomentosa nicely from other related species. The report is well written and informative. I have only few minor suggestions to make.

 Background:

 Besides Pollenia amentaria, also P. atramentaria and P. vera are uniformly black species.

 I doubt that there is any evidence that Pollenia act as disease vectors. They do not really visit feces, cadavers or would be attracted to humans.

 Methdos:

 The sexes of the specimens are not mentioned? The one depicted in the Figure 1 (idPolGris2) is a male. As muscomorph fly males should X and Y chromosomes, I wonder why the latter has not been detected? Can this be influenced by the fact that the Hi-C chromosome contact mapping was performed with another individual (idPolGris1), which might have been a female?

 Overall, the presented genome assembly is yet another nice addition to the growing number of oestroid fly reference genome assemblies, which will hopefully be a nice future resource for any molecular work with these groups.

Are sufficient details of methods and materials provided to allow replication by others?

Yes

Is the rationale for creating the dataset(s) clearly described?

Yes

Are the datasets clearly presented in a useable and accessible format?

Yes

Are the protocols appropriate and is the work technically sound?

Yes

Reviewer Expertise:

molecular biology; evolution; genetics; genomics; Diptera; taxonomy

I confirm that I have read this submission and believe that I have an appropriate level of expertise to confirm that it is of an acceptable scientific standard.

# Reviewer report: Reviewer response for version 1

**DOI:** 10.21956/wellcomeopenres.26065.r126016

**Published:** 2025-07-21

**Authors:** Benoit Nabholz

**Affiliations:** 1 Universite de Montpellier, Montpellier, France

The manuscript presents the genome of *Pollenia griseotomentosa* (Jacentkovský, 1944), produced by the Darwin Tree of Life (DToL) project. Currently, genomes are available for five *Polleniidae* species, all generated by the DToL. These genomes are relatively similar, each with six chromosomes and an approximate size of 1 Gb. I was surprised to read in the introduction that, despite its genome now being fully sequenced, *P. griseotomentosa* remains a poorly known species, with its egg, larva, and host still unknown.

 Once again, this is a high-quality and fully accessible genome produced by this remarkable project.

**Comments:**

- The number of protein-coding genes is currently unknown, as the genome has not yet been annotated despite RNA-seq availability. It is surprising that gene annotations have been presented in genome notes for some species (e.g., *Pollenia angustigena* and *Pollenia amentaria*), but not for others. Some consistency across species would be welcome.
- DNA barcoding was used to confirm species identification, but this is only mentioned in the Methods section. No results are provided, and there is no link to the COI sequences. Including this information would strengthen the manuscript.

Are sufficient details of methods and materials provided to allow replication by others?

Yes

Is the rationale for creating the dataset(s) clearly described?

Yes

Are the datasets clearly presented in a useable and accessible format?

Yes

Are the protocols appropriate and is the work technically sound?

Yes

Reviewer Expertise:

Molecular evolution, Diptera, Hymenoptera, Lepidoptera

I confirm that I have read this submission and believe that I have an appropriate level of expertise to confirm that it is of an acceptable scientific standard.
